# From leadership to innovation: the psychological pathways of intrinsic motivation and creative process engagement

**DOI:** 10.3389/fpsyg.2026.1860466

**Published:** 2026-09-29

**Authors:** Jianqiang Zhang, Chenglei Song

**Affiliations:** 1 School of Accounting, Anhui Wenda University of Information Engineering, Hefei, China; 2 School of Management, Xinyang Agriculture and Forestry University, Xinyang, China

**Keywords:** creative process engagement, employee innovative behavior, intrinsic motivation, responsible leadership, self-determination theory, social information processing theory

## Abstract

**Introduction:**

Although responsible leadership (RL) has attracted increasing attention from the academic community, the correlation mechanism between it and employee innovative behavior (EIB) has not been fully clarified. Based on the data of high-tech enterprises in China, this study explores how intrinsic motivation (IM) and creative process engagement (CPE) explain the relationship between responsible leadership and innovative behavior.

**Method:**

This study uses SPSS 27.0 and AMOS software for statistical analysis. The data is collected on the Credamo platform. After routine data screening and cleaning, 344 valid replies are used for follow-up analysis.

**Result:**

The results showed that RL was positively related to EIB, with IM and CPE independently mediating this relationship. Besides, the sequential indirect effect of RL on EIB through IM and CPE was also significant.

**Discussion:**

The findings indicated that IM and CPE serve as two interconnected mechanisms linking RL with EIB. Drawing on self-determination theory and social information processing theory, this study further illustrates how employees interpret responsible leadership-related information, develop IM, and remain engaged in creative activities.

## 1 Introduction

Responsible Leadership (RL) has gained increasing attention in organizational research in recent years. Faced with the challenge of balancing economic goals with the needs of employees, customers, communities, and other stakeholders, organizations increasingly need to consider the broader impacts of leadership behaviors. Unlike traditional leadership approaches that emphasize organizational performance or leader–employee relationships, RL places greater emphasis on stakeholder participation, ethical decision-making, inclusive communication, and long-term development (Voegtlin, 2011; Miska et al., 2013; Voegtlin et al., 2020). These characteristics are particularly relevant to high-tech enterprises, where employees are required to continuously generate and apply new ideas amid rapid technological changes and complex task demands. Employee Innovative Behavior (EIB) refers to employees’ behaviors of proposing, promoting, and implementing novel ideas and solutions in the workplace, which plays an important role in helping organizations adapt to changes and maintain competitive advantages (Scott and Bruce, 1994). Therefore, how RL is linked to EIB remains an issue requiring further investigation.

Previous studies have linked RL to work engagement, organizational commitment, job satisfaction, voluntary green behavior, and turnover-related outcomes (Voegtlin, 2011; Haque et al., 2019b; Yasin, 2021; Dong and Zhong, 2022; Ahmed et al., 2024). RL may also promote EIB through responsibility awareness, organizational pride, and socially responsible human resource management practices (Su and Lin, 2019; Dong and Zhong, 2021). However, three gaps remain. First, existing research has mainly adopted social exchange, social learning, and social identity perspectives, with limited attention to employees’ internal motivational processes. Although intrinsic motivation (IM) is an important antecedent of innovative behavior (Yeap, 2024; Xu et al., 2023), its mediating role between RL and EIB remains insufficiently examined. Second, prior studies have focused more on innovation outcomes than on how employees translate motivation into innovative action. Creative process engagement (CPE) may represent such a process mechanism (Zhang and Bartol, 2010; Mahmood et al., 2019), but its role between RL and EIB remains underexplored. Finally, IM and CPE have generally been examined separately, despite evidence that IM promotes employees’ participation in creative activities (Dysvik and Kuvaas, 2013). It therefore remains unclear whether RL influences EIB through the sequential mediating roles of IM and CPE.

To address these gaps, this study examines a sequential process through which RL is associated with EIB. Specifically, this study considers IM and CPE as two related mechanisms, suggesting that RL may first enhance employees’ IM and then encourage their involvement in creative activities. By examining this process, this study provides a better understanding of how leadership influences EIB. IM and CPE may represent two connected but distinct stages in the development of EIB. Innovation often involves uncertainty and complex problem-solving. Employees may therefore need to maintain effort even when immediate external rewards are unavailable. IM encourages employees to engage in creative activities because they perceive these activities as meaningful and intrinsically valuable (Amabile et al., 1994). Supportive work environments can further satisfy employees’ psychological needs and strengthen their internal motivation (Paolillo et al., 2021; Xue et al., 2022). Employees with higher IM are also more likely to maintain cognitive flexibility, persist in challenging tasks, and develop original ideas (Chen et al., 2013; Hoang et al., 2023). However, having motivation alone may not be sufficient for innovation. Employees must also invest cognitive and behavioral effort throughout the creative process. CPE reflects employees’ involvement in activities such as problem identification, information searching, evaluating alternative perspectives, and generating solutions (Zhang and Bartol, 2010). Previous studies have connected CPE with leadership behaviors and innovation-related outcomes (Mahmood et al., 2019; Chen et al., 2022). Together, IM and CPE capture two related but distinct aspects of innovation: employees’ internal motivation to innovate and their active engagement in creative activities.

To understand how RL is related to EIB, this study draws on self-determination theory (SDT) and social information processing theory (SIP). These perspectives explain different aspects of employees’ responses to leadership. SIP highlights how employees interpret information conveyed by RL in their work environment, whereas SDT explains how supportive conditions may strengthen employees’ IM (Salancik and Pfeffer, 1978; Deci and Ryan, 2000; Deci et al., 2017). Together, these perspectives link employees’ perceptions of leadership-related cues with the motivational and creative processes involved in EIB. This integrated view helps explain how RL may be associated with EIB through IM and CPE.

Accordingly, this study investigates the relationship between RL and EIB using data from full-time employees in Chinese high-technology enterprises. It focuses on employees’ individual psychological and behavioral responses by examining IM and CPE as separate and sequential mediating mechanisms. The study contributes to existing research in several ways. First, it sheds light on how RL is associated with EIB by revealing the motivational and behavioral processes involved in this relationship. Second, it clarifies the distinction between employees’ willingness to innovate and their involvement in creative activities by considering IM and CPE within the same research framework. Third, it brings together SDT and SIP to explain how leadership-related cues may be linked with employees’ motivation, creative-process involvement, and EIB.

## 2 Hypotheses development

### 2.1 Theoretical framework

SDT and SIP together provide a useful basis for understanding why RL may influence employees’ innovative behavior. SIP suggests that employees do not simply respond to leadership behaviors directly; instead, they interpret leaders’ decisions, interactions, and practices as information about which behaviors are encouraged and valued in the workplace (Salancik and Pfeffer, 1978). Within an RL context, practices such as employee consultation, empowerment, respect, and stakeholder-oriented decision-making may convey signals that support participation, independent judgment, and creative exploration.

These contextual signals can be further explained through SDT. When employees perceive a supportive work environment, their needs for autonomy, competence, and relatedness are more likely to be satisfied, which may facilitate the development of IM (Deci and Ryan, 2000; Deci et al., 2017). Stronger IM may subsequently encourage employees to devote greater cognitive and behavioral effort to CPE, enabling innovative intentions to be translated into EIB. Based on these theoretical arguments, the following hypotheses are developed.

### 2.2 RL and EIB

EIB refers to the extent to which an employee transforms novel ideas, thinking patterns, and problem-solving methods derived from work experience into observable actions (Scott and Bruce, 1994). Existing research has identified leadership style as a key antecedent for shaping creativity and innovative behavior in employees (Carmeli et al., 2013). Specifically, Lin (2023) found that transformational leadership stimulates EIB by establishing good social interactions with subordinates; Zhang and Bartol (2010) pointed out that empowering leaders promote innovative behavior by enhancing the psychological autonomy of their subordinates. Both of these leadership styles fall within the category of positive leadership, and the core logic of their promotion of EIB lies in the empowerment and relational support provided by leaders to their subordinates. RL also has this orientation. Through equal dialogue and consultation with stakeholders, leaders help create an open and inclusive organizational atmosphere, which in turn provides favorable conditions for EIB. Based on this, the study suggests that RL is positively associated with EIB.

RL extends the scope of leader–follower relationships from the traditional hierarchical binary structure to a broader network of stakeholders both within and outside the organization, emphasizing the establishment of trust-based multi-party relationships and recognizing the equal status of stakeholders (Pless and Maak, 2011; Miska and Mendenhall, 2018). When employees’ needs and their status as stakeholders of the organization are sincerely recognized and respected, they are more inclined to respond through greater creative engagement. Furthermore, RL can provide substantial support to employees and safeguard their core interests, thereby strengthening affective commitment (Voegtlin, 2011; Haque et al., 2019a). Employees who demonstrate a strong organizational commitment tend to experience a more positive emotional state, which in turn contributes to the generation of innovative behavior (Munoz et al., 2023). Based on this, we propose the following hypothesis:

H1: RL is positively associated with EIB.

### 2.3 Mediating role of IM

IM refers to an individual’s engagement in activities driven by self-determination and developmental needs rather than by external incentives or contingencies (Deci and Ryan, 1985), and intrinsically motivated individuals tend to show greater initiative and self-direction. SDT suggests that autonomy, competence, and relatedness are the three fundamental psychological needs of an individual (Deci et al., 2017), and when these needs are effectively satisfied, IM is likely to increase (Deci and Ryan, 2000). By helping to satisfy these needs, leadership becomes a key situational factor influencing employees’ IM (Xue et al., 2022), and this relationship has received empirical support in studies of transformational and servant leadership (Al Harbi et al., 2019; Hartnell et al., 2023). First, RL helps to enhance employees’ sense of autonomy by encouraging their participation in work-related decision-making and strengthening their psychological empowerment (Voegtlin, 2011; Doh and Quigley, 2014). In addition, RL can strengthen employees’ sense of competence by recognizing their skills, knowledge, and abilities and supporting their career development (Maak and Pless, 2006; Pless et al., 2012). Finally, by establishing equal and trust-based relationships, RL helps employees feel respected and supported, thereby satisfying their need for relatedness (Pless and Maak, 2011). Together, these practices suggest that RL can strengthen employees’ IM.

Prior research has also examined IM as a mediating mechanism linking positive leadership or work contexts to employee outcomes. Al Harbi et al. (2019) examined psychological empowerment and IM as mechanisms through which transformational leadership influences employee creativity. Hartnell et al. (2023) showed that IM helps explain the relationship between servant leadership and employee performance. Xu et al. (2023) further identified IM as part of a chain-mediating process linking a perceived innovation-oriented human resource system to innovative work behavior. Although these studies did not directly examine RL, they provide empirical grounds for treating IM as a mediator between supportive leadership or organizational conditions and innovation-related outcomes.

Regarding how IM influences EIB, individuals with high levels of IM tend to have stronger risk tolerance and cognitive flexibility (Koestner and Losier, 2002) and are more likely to generate original ideas and put them into practice. This intrinsic drive is also closely associated with sustained persistence in the face of obstacles (Al Harbi et al., 2019; Hoang et al., 2023), enabling employees to challenge the status quo and proactively seek creative solutions (Zhang and Zhou, 2014). Empirical studies have also identified a positive relationship between IM and EIB (Chen et al., 2013; Hoang et al., 2023). Based on the above reasoning, the following hypothesis is proposed:

H2: IM mediates the relationship between RL and EIB.

### 2.4 Mediating role of CPE

CPE refers to employees’ involvement in key stages of the creative process, including problem identification, information search and encoding, and the development of novel solutions (Reiter-Palmon and Illies, 2004; Zhang and Bartol, 2010). Through participation in these activities, employees devote cognitive and behavioral resources to exploring, developing, and refining creative ideas. Previous research has linked CPE with several leadership styles, such as transformational leadership, empowering leadership, and servant leadership (Zhang and Bartol, 2010; Mahmood et al., 2019; Chen et al., 2022; López-Lara and Arana-Barbier, 2026). However, whether RL can similarly facilitate employees’ engagement in creative processes remains less explored.

SIP theory provides a useful perspective for understanding this relationship because employees interpret leadership behaviors as information about what types of attitudes and actions are valued in their work environment (Salancik and Pfeffer, 1978; Meyer, 1994). Through employee involvement in decision-making and the provision of greater autonomy, RL may communicate that participation and creative exploration are encouraged (Doh and Quigley, 2014). In addition, RL emphasizes relationships characterized by trust, respect, and equality between leaders and employees (Stahl and Sully de Luque, 2014). Such relational experiences may encourage employees to participate more actively in exploratory and creative activities, increasing their willingness to remain engaged in the creative process (Mueller and Kamdar, 2011).

Prior research has examined CPE as an intervening mechanism linking leadership-related conditions with creative and innovative outcomes. Zhang and Bartol (2010) incorporated CPE into the process through which empowering leadership influenced employee creativity. Henker et al. (2015) showed that CPE formed part of the sequential mechanism linking transformational leadership with employee creativity, while Saeed et al. (2019) identified CPE as a mechanism linking leader-member exchange with innovative work behavior. Together, these findings indicate that CPE can transmit leadership-related influences to creative and innovative outcomes.

CPE may further contribute to EIB by directing employees’ attention and effort toward the development of workable ideas. Through problem identification, information search, and the exploration of alternative solutions, employees can refine initial ideas and improve their novelty and feasibility (Reiter-Palmon and Illies, 2004; Zhang and Bartol, 2010). Accordingly, RL may be associated with EIB by encouraging employees to engage more deeply in the creative process.

H3: CPE mediates the relationship between RL and EIB.

### 2.5 Sequential mediating roles of IM and CPE

IM and CPE represent interrelated but functionally distinct stages in the development of EIB. IM reflects employees’ internally driven willingness to engage in innovative work, whereas CPE represents their cognitive and behavioral involvement in problem identification, information search, and solution generation (Amabile et al., 1994; Reiter-Palmon and Illies, 2004; Zhang and Bartol, 2010). Although employees may be willing to innovate, such willingness does not necessarily result in sustained engagement in the activities required to develop and implement new ideas.

As argued in H2, RL may strengthen employees’ IM. Employees with stronger IM are more likely to invest attention and cognitive resources in challenging tasks, persist when solutions are not immediately available, and explore alternative approaches. These motivational tendencies may increase their involvement in CPE (Zhang and Bartol, 2010; Azim et al., 2019). CPE, in turn, helps employees refine initial ideas, evaluate their feasibility, and transform them into implementable innovative solutions. Thus, IM may provide the willingness to innovate, whereas CPE represents the process through which this willingness is enacted.

This ordering is consistent with the integrated framework, in which RL-related contextual cues may be internalized as motivation and subsequently enacted through creative-process involvement. Previous studies have provided related evidence for an ordered relationship between motivational states and creative-process engagement (Zhang and Bartol, 2010; Henker et al., 2015). Leadership research has also used sequential mediation models to explain innovative behavior through ordered contextual and cognitive mechanisms (Malibari and Bajaba, 2022). However, the specific RL-IM-CPE-EIB pathway has not been directly examined. Therefore, extending previous research, this study proposes that RL may be associated with EIB through the sequential roles of IM and CPE.

Accordingly, we propose:

H4: IM and CPE sequentially mediate the relationship between RL and EIB.

Based on the theoretical analysis presented above, the conceptual research model is depicted in Figure 1.

**FIGURE 1:**
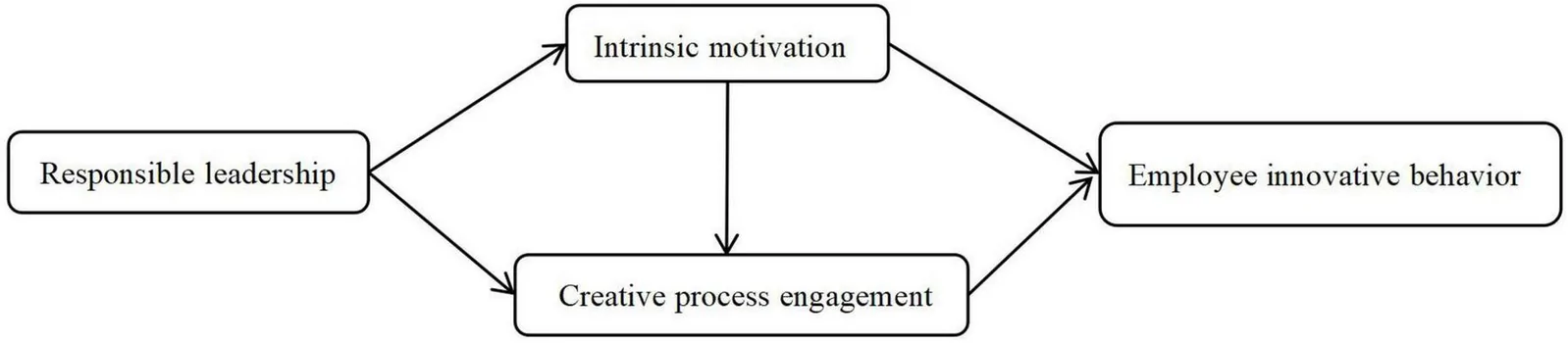
The conceptual research model.

## 3 Materials and methods

### 3.1 Sample and procedure

The survey was distributed online through the Credamo platform to full-time employees working in Chinese high-technology enterprises between February and March 2026. The high-technology context was considered appropriate because employees in this sector are more likely to encounter technology-driven changes and innovation-related work practices (Gao and Liu, 2021). Prior to participation, respondents were informed that the survey was conducted solely for academic purposes and that their personal information would not be disclosed. Data collection followed a three-stage procedure with a 2-week interval between each stage. During the first stage, 490 employees were invited to complete a survey containing demographic information and RL measures, and 469 usable responses were obtained. After a 2-week interval, the second-stage survey was administered to these participants to assess IM, yielding 437 valid responses. Two weeks later, the final survey was administered to assess CPE and EIB, resulting in 419 completed questionnaires. A unique code was assigned to each participant to link responses across the three stages while preserving anonymity. Respondents who were unable to continue participation due to resignation, leave, or other reasons were not included in later stages. Following the screening process, questionnaires showing low response quality, such as identical answering patterns or extremely short completion times (Ward and Meade, 2023), were removed. The final dataset consisted of 344 valid responses, corresponding to an effective response rate of 82.1%.

Of the 344 samples, 51.2% were female; The 30–39 age group accounted for 50.6 percent; 44.6 percent of respondents have worked at their current workplace for 6–10 years; The highest educational attainment was a bachelor’s degree (44.6 percent).

### 3.2 Measures

All measurement scales employed in this study are established scales that have been applied in domestic and international research. To account for potential language differences, we adopted a “translation-retranslation” process to ensure measurement quality. All items were scored using a five-point Likert scale, with ratings ranging from 1 (strongly disagree) to 5 (strongly agree).

First, the single-dimensional five-item scale developed by Voegtlin (2011) was adopted to measure RL. A representative item is: “My leader demonstrates awareness of the relevant stakeholder claims” (Cronbach’s α = 0.860). Next the scale developed by Guay et al. (2000) with 4 items was used to measure IM. A representative item is: “Because this activity is fun” (Cronbach’s α = 0.818). Then CPE was measured using the three-dimensional, 11-item scale originally developed by Reiter-Palmon and Illies (2004) and subsequently adapted by Zhang and Bartol (2010). A representative item is: “I think about the problem from multiple perspectives” (Cronbach’s α = 0.731). Finally a unidimensional six-item scale developed by Scott and Bruce (1994) was employed to measure EIB. A representative item is: “I consistently generate creative ideas” (Cronbach’s α = 0.872). Furthermore, consistent with prior research on innovative behavior (Han et al., 2023; Li et al., 2020; Javed et al., 2019), gender, age, organizational tenure, and educational attainment were included as control variables.

## 4 Results

### 4.1 Confirmatory factor analysis (CFA)

Confirmatory factor analysis of the primary study variables was conducted using AMOS software; results are presented in Table 1. The hypothesized four-factor model demonstrated superior fit relative to all alternative nested models (χ^2^/df = 2.172, CFI = 0.909, IFI = 0.910, RMSEA = 0.058, SRMR = 0.054) providing evidence that the four variables are conceptually distinct and exhibit satisfactory discriminant validity.

**TABLE 1:** Model fit (*N* = 344).

Model	χ 2	df	χ 2/df	CFI	IFI	RMSEA	SRMR
Four-factor model (RL, IM, EIB, CPE)	616.936	284	2.172	0.909	0.910	0.058	0.054
Three-factor model (RL+IM, EIB, CPE)	755.041	287	2.631	0.872	0.873	0.069	0.076
Two-factor model (RL+IM+EIB, CPE)	971.392	289	3.361	0.813	0.814	0.083	0.072
Single-factor model (RL+IM+EIB+CPE)	1076.419	290	3.712	0.784	0.786	0.089	0.077

+ = Two factors were combined; RL = responsible leadership, IM = intrinsic motivation; CPE = creative process engagement, EIB = employee innovative behavior.

### 4.2 Common method variance

Because all variables in this study were assessed via employee self-report, potential CMB warranted examination. Harman’s single-factor test was conducted to evaluate its impact. An unrotated exploratory factor analysis of all measurement items produced multiple factors with eigenvalues greater than 1. The first factor explained 31.699% of the total variance, which was below the 40% criterion, suggesting that common method bias was unlikely to be a serious concern. To further examine potential common method bias, we introduced a common latent method factor into the four-factor measurement model and allowed all items to load on this factor (Podsakoff et al., 2003). The resulting model showed CFI = 0.948, IFI = 0.949, RMSEA = 0.045, and SRMR = 0.038. Compared with the original four-factor model (CFI = 0.909, IFI = 0.910, RMSEA = 0.058, SRMR = 0.054), the changes were ΔCFI = 0.039, ΔIFI = 0.039, ΔRMSEA = 0.013, and ΔSRMR = 0.016. As these differences remained below the adopted cutoffs of 0.10 for CFI and IFI and 0.05 for RMSEA and SRMR, the addition of the method factor did not substantially improve model fit. Common method bias therefore does not appear to be a major concern in this study.

### 4.3 Descriptive statistics and correlation analysis

Table 2 shows that all primary study variables were significantly and positively intercorrelated, consistent with theoretical expectations. CPE is significantly and positively associated with EIB (*r* = 0.662, *p* < 0.01), exceeding 0.6, raising the possibility of multicollinearity. However, variance inflation factors (VIF) calculated for each variable yielded a maximum VIF of 2.041, substantially below the critical cutoff of 5, confirming that multicollinearity is not a concern and that the data are suitable for subsequent hypothesis testing.

**TABLE 2:** Mean, standard deviation and correlation coefficient of variables (*N* = 344).

Variables	M	SD	1	2	3	4	5	6	7
1. Gender	1.510	0.501	0.149**	0.715**	0.278**	0.032	0.505**	0.572**	0.662**
2. Age	3.120	0.800							
3. Tenure	3.230	0.886	0.118*						
4. Edu	3.030	0.877	0.032	0.348**					
5. RL	4.012	0.631	0.008	−0.065	−0.060				
6. IM	4.214	0.471	0.025	−0.045	−0.052	−0.016			
7. CPE	4.164	0.438	−0.011	−0.074	−0.102	0.049	0.477**		
8. EIB	4.111	0.497	0.044	−0.041	−0.096	0.035	0.424**	0.596**	

*p < 0.05, **p < 0.01.

### 4.4 Hypothesis testing

As shown in Table 3, Model 3 indicates that RL was positively associated with EIB (*b* = 0.42, *p* < 0.001), supporting H1. After IM was included in Model 4, the coefficient of RL remained positive and significant but was smaller than that in Model 3 (*b* = 0.161, *p* < 0.01), consistent with partial mediation and supporting H2. Similarly, Model 5 shows that after introducing CPE, the direct effect of RL on EIB was also weakened (*b* = 0.141, *p* < 0.01), consistent with partial mediation by CPE and supporting H3.

**TABLE 3:** Summary of the hierarchical regression results (*N* = 344).

Variables	Intrinsic motivation	Creative process engagement	Employees innovative behavior		
	Model 1	Model 2	Model 3	Model 4	Model 5
Gender	0.022	−0.028	0.035	0.024	0.052
Age	0.019	−0.005	0.071	0.061	0.074
Tenure	−0.023	−0.09	−0.126	−0.114	−0.073
Edu	−0.034	0.062	0.03	0.048	−0.006
RL	0.506***	0.469***	0.42***	0.161**	0.141**
IM				0.511***	
CPE					0.594***
R^2^	0.257	0.237	0.19	0.384	0.459
F	23.349***	20.970***	15.849***	35.021***	47.716***

**p < 0.01, ***p < 0.001.

To further validate H2, H3, and H4, this study employed SPSS process macros (Model 6; Preacher and Hayes, 2008) for bootstrap-based mediation analysis, which included 5000 bootstrap resamples and 95% bias-corrected confidence intervals. As shown in Table 4, the indirect effect of RL on EIB via IM was statistically significant (*b* = 0.122, 95% CI [0.055, 0.188], excluding 0), further supporting H2. Besides, the mediated effect value of CPE was likewise significant (*b* = 0.089, 95% CI [0.029, 0.151], excluding 0), supporting H3. The sequential indirect effect through IM and CPE in tandem yielded an effect estimate of 0.081 (95% CI [0.040, 0.143], excluding 0), supporting H4. In sum, all hypotheses received empirical support.

**TABLE 4:** Summary of the mediation analysis results (*N* = 344).

Relationship	Effect	SE	BootLLCI	BootULCI
Total indirect effect	0.292	0.041	0.212	0.374
RL-IM-EIB	0.122	0.034	0.055	0.188
RL-CPE-EIB	0.089	0.032	0.029	0.151
RL-IM-CPE-EIB	0.081	0.027	0.040	0.143

## 5 Discussion

The research results show that employees’ responses to RL involve both motivational and behavioral processes. This shows that the relationship between RL and EIB may be a gradual development process, that is, employees first form the intrinsic motivation to participate in innovative activities, and then translate this motivation into active investment in creative activities. Therefore, the results of this study further deepen the understanding of how RL is associated with EIB through the psychological and behavioral responses of employees.

In addition, the results of this study support the integration of SDT and SIP to explain the psychological process behind the relationship between RL and EIB. From the perspective of SIP, employees may interpret information related to RL in the work environment and form a perception of whether the working environment can provide support and participation opportunities. SDT further explains how these experiences are linked to employees’ IM and subsequent innovation-related behaviors. This motivational and behavioral process may be particularly relevant in the context of China’s high-tech enterprises, where employees often face rapid technological changes, complex problem-solving requirements and continuous innovation demands. Under such conditions, employees’ innovative behavior depends not only on their willingness to generate new ideas, but also on their sustained involvement in creative activities. Therefore, RL-related supportive experiences may play an important role in enhancing employee IM and promoting CPE. In general, SDT and SIP provide a complementary theoretical perspective for understanding employees’ motivational and behavioral processes in the RL-EIB relationship.

### 5.1 Theoretical and empirical implications

Previous studies have revealed the significant impact of leadership behavior on EIB. However, the mechanisms underlying the relationship between RL and EIB remain insufficiently explored. We incorporated IM and CPE into the same analytical framework, moving beyond the previous research focus on the direct relationship between RL and EIB, thereby further revealing how leadership-related contexts influence employees’ innovation motivation and creative engagement.

Furthermore, this study deepens the understanding of how RL is related to EIB by distinguishing the different roles of IM and CPE in the innovation process and their internal connections. IM reflects the intrinsic motivation of employees to participate in innovative activities, while CPE represents the process through which employees subsequently engage in creative activities. Rather than considering the two as independent explanatory mechanisms, this study views IM and CPE as interconnected components of a continuous process. This perspective differs from prior leadership and innovation studies that have mainly examined psychological and behavioral mechanisms as separate explanations of innovative outcomes. By integrating these two mechanisms, this study highlights how leadership-related experiences are progressively translated from employees’ internal motivation into active involvement in creative activities. The significant indirect effects of IM and CPE, as well as the indirect effects of RL acting through IM and CPE on EIB, provide further support for this mechanism. Overall, this perspective provides a more process-oriented explanation of how RL-related experiences are transformed into EIB through employees’ motivational and behavioral processes.

Finally, this study integrates SDT and SIP to further explain the mechanism linking RL and EIB. SIP emphasizes how employees interpret RL-related cues from their work environment, whereas SDT explains how such contextual experiences may be internalized as IM through the satisfaction of psychological needs. Integrating these perspectives helps clarify how RL-related contextual information shapes employees’ motivation and subsequently relates to CPE and EIB.

### 5.2 Practical implications

This study offers several practical implications for organizations.

First, RL plays a crucial role in facilitating EIB. Managers should not only focus on the innovation goals themselves, but also pay attention to the experiences and feelings of employees during the work process. For instance, managers can help employees develop a more positive perception of their work environment by listening to their opinions, providing necessary support, and recognizing their efforts and contributions in the innovation activities. In turn, employees may tend to participate actively in innovation-related tasks and demonstrate higher levels of EIB.

Second, organizations should create work conditions that strengthen employees’ autonomy, competence, relatedness, and sense of meaningful work, rather than relying solely on material rewards or external pressure to encourage employee innovation. Several methods can be considered to enhance employees’ IM and encourage their involvement in innovation-related tasks, such as providing greater decision-making autonomy, opportunities for skill development, and meaningful work experiences.

Furthermore, managers should pay attention to employees’ continuous involvement in the innovation process. Managers can create a more open innovation climate by encouraging idea sharing, facilitating the exchange of diverse viewpoints, and providing opportunities for employees to solve practical problems. These practices may enhance employees’ CPE and encourage deeper involvement in creative activities. Such continuous engagement can help employees further refine innovative ideas and translate them into actual EIB.

### 5.3 Limitations and future research

This study has several limitations that need to be acknowledged. All the core variables are based on self-reports. Therefore, even though data were collected at multiple time points and subjected to the Harman single-factor test and common method factor analysis, the residual influence of common method bias still cannot be completely eliminated. Moreover, the research design provides relatively limited support for causal inference because the temporal sequence among the variables has not been fully established. Future research can address these issues by introducing multi-source data (such as superior evaluations or colleague evaluations) and adopting longitudinal or experimental designs.

Second, high-tech enterprises usually encounter numerous tasks related to technological changes and innovation. Therefore, this paper selects this industry as the research object, providing a relatively appropriate research background for exploring the relationship between RL and EIB. However, since the samples are from a single industry, the applicability of the research conclusions in other industries or organizational environments still needs to be further tested. Therefore, future research can expand the sample sources and include different types of industries and organizations to further examine the applicability of this model in different situations.

Furthermore, this study did not include the job information of the participants, thus failing to examine the potential differences in the relationships between RL, IM, CPE and EIB among employees in different positions. Future research could combine the employees’ career backgrounds and job characteristics to conduct further analysis of the mechanism at play under different job responsibilities.

In addition, the theoretical account of the RL–EIB relationship in this study rests primarily on a social psychological perspective. While this perspective is useful for explaining employees’ psychological reactions to leadership, alternative theoretical lenses, such as social network theory or a resource-based view, may offer further insight into the mechanisms through which RL relates to individual innovative behavior.

## Data Availability

The data presented in this study are available on reasonable request from the corresponding author.
